# Towards the automatic detection of skin lesion shape asymmetry, color variegation and diameter in dermoscopic images

**DOI:** 10.1371/journal.pone.0234352

**Published:** 2020-06-16

**Authors:** Abder-Rahman Ali, Jingpeng Li, Sally Jane O’Shea

**Affiliations:** 1 Division of Computer Science and Mathematics, University of Stirling, Stirling, Scotland, United Kingdom; 2 Mater Private Hospital, Dublin, Ireland; University of Queensland Diamantina Institute, AUSTRALIA

## Abstract

Asymmetry, color variegation and diameter are considered strong indicators of malignant melanoma. The subjectivity inherent in the first two features and the fact that 10% of melanomas tend to be missed in the early diagnosis due to having a diameter less than 6mm, deem it necessary to develop an objective computer vision system to evaluate these criteria and aid in the early detection of melanoma which could eventually lead to a higher 5-year survival rate. This paper proposes an approach for evaluating the three criteria objectively, whereby we develop a measure to find asymmetry with the aid of a decision tree which we train on the extracted asymmetry measures and then use to predict the asymmetry of new skin lesion images. A range of colors that demonstrate the suspicious colors for the color variegation feature have been derived, and Feret’s diameter has been utilized to find the diameter of the skin lesion. The decision tree is 80% accurate in determining the asymmetry of skin lesions, and the number of suspicious colors and diameter values are objectively identified.

## Introduction

Melanoma can be recognizable by most physicians and patients when in its advanced stage. However, it is highly curable if diagnosed early and treated timely [1–2]. With the advent of immunotherapy and targeted therapy, melanoma treatments have improved significantly; the five-year survival rate of early stage melanoma and metastatic melanoma (i.e. stage IV) is 98.4% and 22.5%, respectively [3]. A group of researchers at the New York University found the ABCD rule (Asymmetry, Border irregularity, Color variegation, and Diameter greater than 6mm) in 1985 as a simple framework that physicians, novice dermatologists and non-physicians could use to learn about the features of melanoma in its early curable stage [4]; the rule is being promoted by the American Cancer Society as a method to help in seeking early medical evaluation of any suspicious pigmented lesions. The ABCD rule provides a checklist of premalignant changes in skin lesions and an appraisal of pigmented cutaneous lesions that may need to be further examined by a specialist which might result in further work of dermoscopy or biopsy, or both.

Skin lesion asymmetry is a strong indicator of malignant melanoma [5] such that the degree of asymmetry displayed by a skin lesion is indicative of its malignant potential. As opposed to benign pigmented skin lesions that are usually circular and symmetric, melanomas tend to develop in an uncontrolled fashion and grow at an irregular rate, rendering them to be asymmetric [6]. In layman terms, asymmetry refers to the fact that when drawing a line through the middle of the mole the two halves will not match, meaning that the shape of one half doesn’t resemble the other half (lopsided in shape), providing a warning sign of melanoma. There is no consensus on what asymmetry extent is required before one can tell that the skin lesion is considered asymmetric. It has been shown that the percentage of disagreement between dermatologists on the presence of asymmetry is around 5-10% [4]. A more objective measurement of asymmetry is thus deemed necessary.

Asymmetry evaluation is carried out by separating the lesion into four sectors using orthogonal axes that pass through the lesion centroid and are aligned so that minimum asymmetry (maximum symmetry) is obtained [7]. Different attempts have been made to automatically determine the asymmetry of skin lesions in literature, which can be summarized as shown in Table 1.

**Table 1:** Determining asymmetry of skin lesions in literature.

1	Measuring asymmetries of skin lesions [8]
2	Determining the asymmetry of skin lesion with fuzzy borders [9]
3	Automatic detection of asymmetry in skin tumors [4]
4	Digital videomicroscopy and image analysis with automatic classification for detection of thin melanomas [10]
5	Digital dermoscopy analysis for the differentiation of atypical nevi and early melanoma [11]
6	Qualitative asymmetry measure for melanoma detection [12]
7	Irregularity and asymmetry analysis of skin lesions based on multi-scale local fractal distributions [13]
8	Determination of optimal axes for skin lesion asymmetry quantification [14]

Color variegation is considered the earliest sign of melanoma, and has a high predictability for the diagnosis of the disease. In fact, studies have demonstrated that color variegation might be the most important singular discriminator of melanoma [15]. It refers to the presence of two or more shades of pigment (two or more colors) within the skin lesion border. As opposed to benign lesions which tend to be generally uniform in color, melanoma lesions tend to often contain more than two colors, meaning that the color composition within the skin lesion is inhomogeneous. Melanoma in particular contains one or more of these six suspicious shades of color: white, red, light brown, dark brown, blue-gray, and black.

To characterize the color composition within lesions different approaches have been proposed in literature as depicted in Table 2.

**Table 2:** Characterizing color variegation in literature.

1	Skin cancer diagnostics with an all-inclusive smartphone application [16]
2	Detection of melanoma from dermoscopic images of naevi acquired under uncontrolled conditions [17]
3	Automatic color segmentation of images with applications in detection of variegated coloring in skin tumors [18]
4	Classification of malignant melanoma and benign skin lesions: implementation of automatic ABCD rule [19]
5	Automated malignant melanoma detection using Matlab [20]

Most early melanomas (i.e. stage 0) tend to be larger than 6mm in diameter (i.e. size of a pencil eraser). Some studies argue that smaller diameters can exist in melanoma, which makes this criterion not absolute especially that 10% of melanomas tend to be missed in the early diagnosis (i.e. diameter <6mm) if the diagnosis was based only on diameter, making it preferable to use a computer vision system when evaluating diameter [21]. Table 3 lists different studies used to find the skin lesion diameter.

**Table 3:** Measuring skin lesion diameter in literature.

1	Simple matlab tool for automated malignant melanoma diagnosis [22]
2	Skin cancer diagnostics with an all-inclusive smartphone application [16]
3	Computer-aided diagnosis of melanoma using border and wavelet-based texture analysis [23]
4	Diagnosis of skin lesions based on dermoscopic images using image processing techniques [24]

The aim of this paper is to present an automated approach to determining skin lesion asymmetry, color variegation, and diameter in dermoscopic images, where different methods and measures are proposed for carrying out this task. Border irregularity (i.e. the B feature in the ABCD rule) has been discussed in our other work in [25].

## Methods

### Image segmentation

In this section we describe the segmentation method utilized in the paper. In particular, we propose an improved version of Otsu’s method [26] for skin lesion segmentation coupled with pre-processing and post-processing stages as described below.

Say we have two classes: lesion (*L*) and skin (*S*), the variance of the pixels in those two classes can be defined as:

$$\begin{array}{c} \sigma_{L}^{2}(k)=\sum_{i=0}^{k-1}{\left(i-\mu_{L}(k)\right)}^{2}\frac{p_{i}}{P_{L}(k)} \end{array}$$ (1)

$$\begin{array}{c} \sigma_{S}^{2}(k)=\sum_{i=k}^{L-1}{\left(i-\mu_{S}(k)\right)}^{2}\frac{p_{i}}{P_{S}(k)} \end{array}$$ (2)

where *k* is the graylevel value, [0, *L* − 1] is the range of graylevel (intensity) levels, *p*_*i*_ is the number of times pixel (graylevel) *i* occurred in the image which can be obtained from the image histogram. The histogram is normalized and perceived as a probability distribution, that is:

$$\begin{array}{c} p_{i}=\frac{n_{i}}{N},p_{i}\geq 0,\sum_{i=0}^{L-1}p_{i}=1 \end{array}$$ (3)

where *n*_*i*_ is the number of pixels at graylevel *i*, and *N* = (*n*_0_+ *n*_1_+ …*n*_*L*_ − 1) represents the total number of pixels in the image.

*μ*_*L*_ and *μ*_*S*_ represent the lesion class mean and skin class mean, respectively, which are defined as:

$$\begin{array}{c} \mu_{L}(k)=\sum_{i=0}^{k-1}\frac{ip_{i}}{P_{L}(k)} \end{array}$$ (4)

$$\begin{array}{c} \mu_{S}(k)=\sum_{i=k}^{L-1}\frac{ip_{i}}{P_{S}(k)} \end{array}$$ (5)

*P*_*L*_ and *P*_*S*_ represent the probabilities of class occurrence of the lesion and skin, respectively, defined as:

$$\begin{array}{c} P_{L}(k)=\sum_{i=0}^{k-1}p_{i} \end{array}$$ (6)

$$\begin{array}{c} P_{S}(k)=\sum_{i=k}^{L-1}p_{i} \end{array}$$ (7)

The *within class variance* which Otsu’s method attempts to minimize by finding an optimal threshold is defined as:

$$\begin{array}{c} \sigma_{W}^{2}(k)=P_{L}(k)\times \sigma_{L}^{2}(k)+P_{S}(k)\times \sigma_{S}^{2}(k) \end{array}$$ (8)

The *between class variance* on the other hand, which Otsu’s method attempts to maximize, is defined as:

$$\begin{array}{c} \sigma_{B}^{2}(k)=P_{L}(k)\times {\left(\mu_{L}(k)-\mu_{T}(k)\right)}^{2}+P_{S}(k)\times {\left(\mu_{S}(k)-\mu_{T}(k)\right)}^{2} \end{array}$$ (9)

where *μ*_*T*_(*k*) is the total mean, defined as:

$$\begin{array}{c} \mu_{T}(k)=P_{L}(k)\times \mu_{L}(k)+P_{S}(k)\times \mu_{S}(k) \end{array}$$ (10)

This is equivalent to Eq 11, that is subtracting the within-class variance from the total variance (*σ*^2^) of the combined distribution.

$$\begin{array}{c} \sigma_{B}^{2}(k)=\sigma^{2}-\sigma_{W}^{2}(k) \end{array}$$ (11)

The threshold *k* with the maximum between-class variance has also the minimum within-class variance.

The class separability *η* is:

$$\begin{array}{c} \eta =\frac{\sigma_{B}^{2}(k)}{\sigma_{W}^{2}(k)} \end{array}$$ (12)

In our proposed improvement, which we refer to here as *Otsu-II*, instead of using *P*_*L*_(*k*) and *P*_*S*_(*k*) we use the new measures *D*_*L*_(*k*) and *D*_*S*_(*k*) defined as:

$$\begin{array}{c} f_{L}(k)=ip_{i};\{i\in R:0<i<k-1\} \end{array}$$ (13)

$$\begin{array}{c} D_{L}(k)=\sigma^{{}^{\prime }}(f_{L}(k)) \end{array}$$ (14)

$$\begin{array}{c} f_{S}(k)=ip_{i};\{i\in R:k<i<L-1\} \end{array}$$ (15)

$$\begin{array}{c} D_{S}(k)=\sigma^{{}^{\prime }}(f_{S}(k)) \end{array}$$ (16)

where *σ*′ is the normalized standard deviation ($\sigma^{{}^{\prime }}=\frac{\sigma_{i}}{N}$). $\sigma_{W}^{2}$ and $\sigma_{B}^{2}$ can be rewritten as:

$$\begin{array}{c} \sigma_{W}^{2}(k)=D_{L}(k)\times \sigma_{L}^{2}(k)+D_{S}(k)\times \sigma_{S}^{2}(k) \end{array}$$ (17)

$$\begin{array}{c} \sigma_{B}^{2}(k)=D_{L}(k)\times {\left(\mu_{L}(k)-\mu_{T}(k)\right)}^{2}+D_{S}(k)\times {\left(\mu_{S}(k)-\mu_{T}(k)\right)}^{2} \end{array}$$ (18)

Thus, how spread out the pixel intensities are in each class is taken into account, such that when the pixel intensities are spread apart *D* will be large and when they are tightly bunched together *D* will be small. Standard deviation is used since it expresses the statistical distribution of each class more accurately than variance, especially that the dispersion of classes are measured as the distance between the mean of a class and any intensity value, which is proportional to the standard deviation rather than variance. This makes the optimal threshold less biased towards the larger variance among two class variances (i.e. larger dispersion of two classes) [27]. Segmentation performance of using the new measures will be demonstrated in terms of the Dice coefficient [28] in the results section.

**Fig 1:**
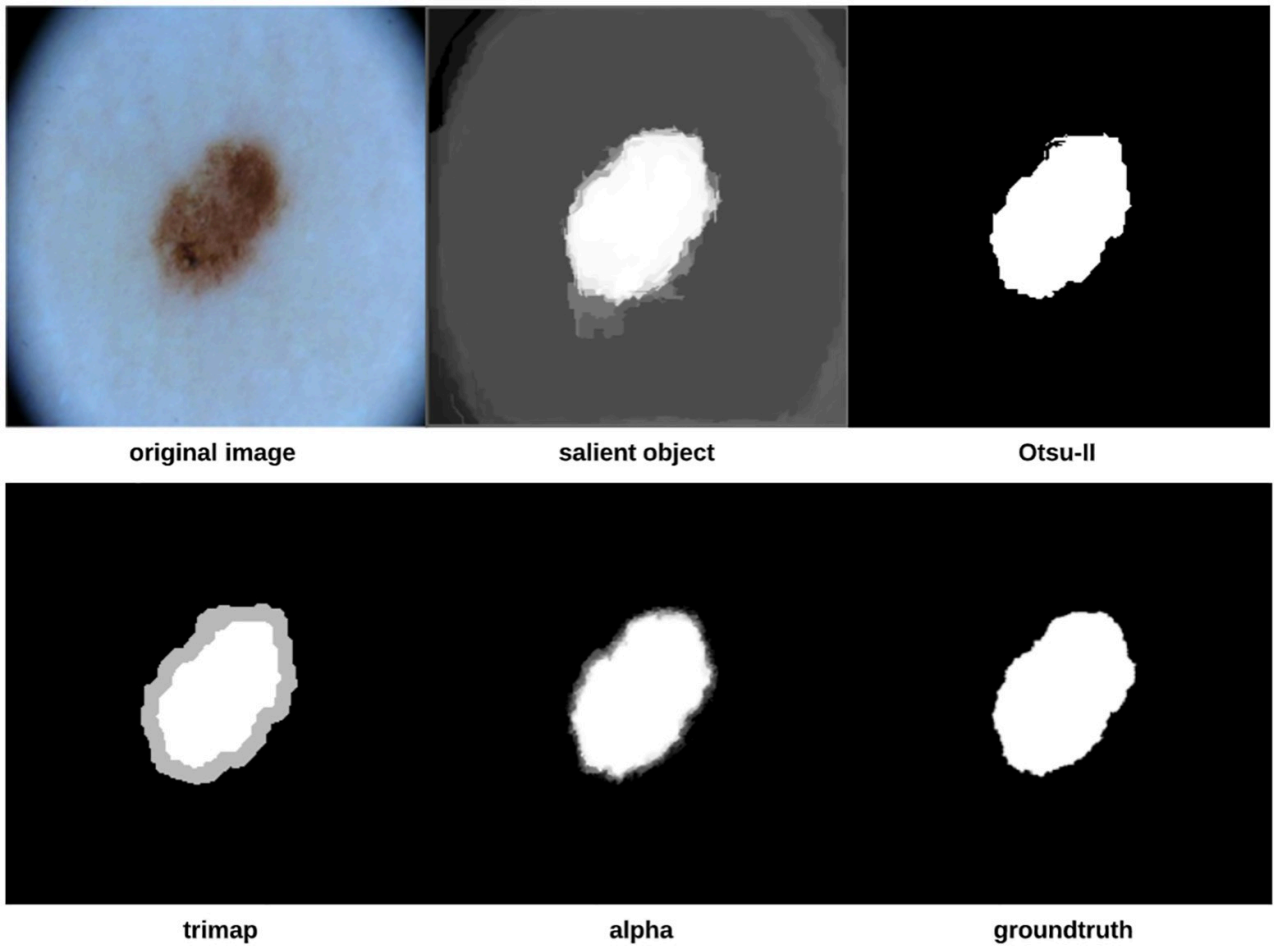
Proposed skin lesion segmentation approach.

To improve the segmentation process further, we introduce pre-processing and post-processing operations which together form the proposed approach depicted in Fig 1. The approach starts by locating the important parts of the image (i.e. lesion). Otsu-II is then applied on the salient object to obtain a binary image that will be used as a groundtruth in the trimap generation step in which each image pixel will be assigned to either the lesion, skin, or as a mixture of both the foreground (lesion) and background (skin). The alpha matte is finally created to distinguish more accurately between the skin and lesion, eventually finding the final segmentation.

*Saliency detection* is the process of automatically locating the important parts of an image, where saliency refers to the unique features (i.e. pixels) of the image. The output of the saliency detection step is a map where the intensity of each pixel represents the probability of the pixel belonging to a salient object. In this work, we use a Discriminative Regional Feature Integration approach (DRFI) for saliency detection [29, 30] as it is considered one of the most efficient algorithms for saliency detection [30].

After detecting the salient objects, we apply our improved Otsu method (i.e. Otsu-II) on the objects to create binary images that will serve as image masks (i.e. groundtruth) in the trimap generation step, in which each pixel in the image is assigned to three possible values: definite foreground (lesion), definite background (skin) and uncertain (a mixture of foreground and background pixels). To create the trimap, erosion and dilation morphological operations are applied on the binary image mask of the skin lesions (i.e. salient objects). The trimap can be generated using the following formula [31]:

$$\begin{array}{c} M(R)=F(E)\cup B\left(D\right)\cup M\left(G\right) \end{array}$$ (19)

where *M*(.) refers to a set of pixels in the image, and *F*(.) and *B*(.) are the functions that extract the foreground and background pixels, respectively. *R*, *E*, *D* and *G* denote the trimap, eroded image, dilated image, and the gap between the foreground and background resulting from the morphological operations, respectively. *F*(*E*) represents the foreground pixels (white), *B*(*D*) represents the background pixels (black), and *M*(*G*) represents the uncertain pixels (gray).

The final step in the proposed approach is matting, that is finding (creating) the alpha matte/channel *α*_*n*_ which is used to accurately distinguish between the foreground and background, rendering the final segmentation. In this step, we utilize KNN (K-nearest Neighbors) matting [32] to find *α*_*n*_, which is represented as follows:

$$\begin{array}{c} \alpha_{n}=KNN\{I_{n},T_{n}\} \end{array}$$ (20)

where the inputs *I*_*n*_ and *T*_*n*_ are the original input image and the corresponding trimap image, respectively.

**Fig 2:**
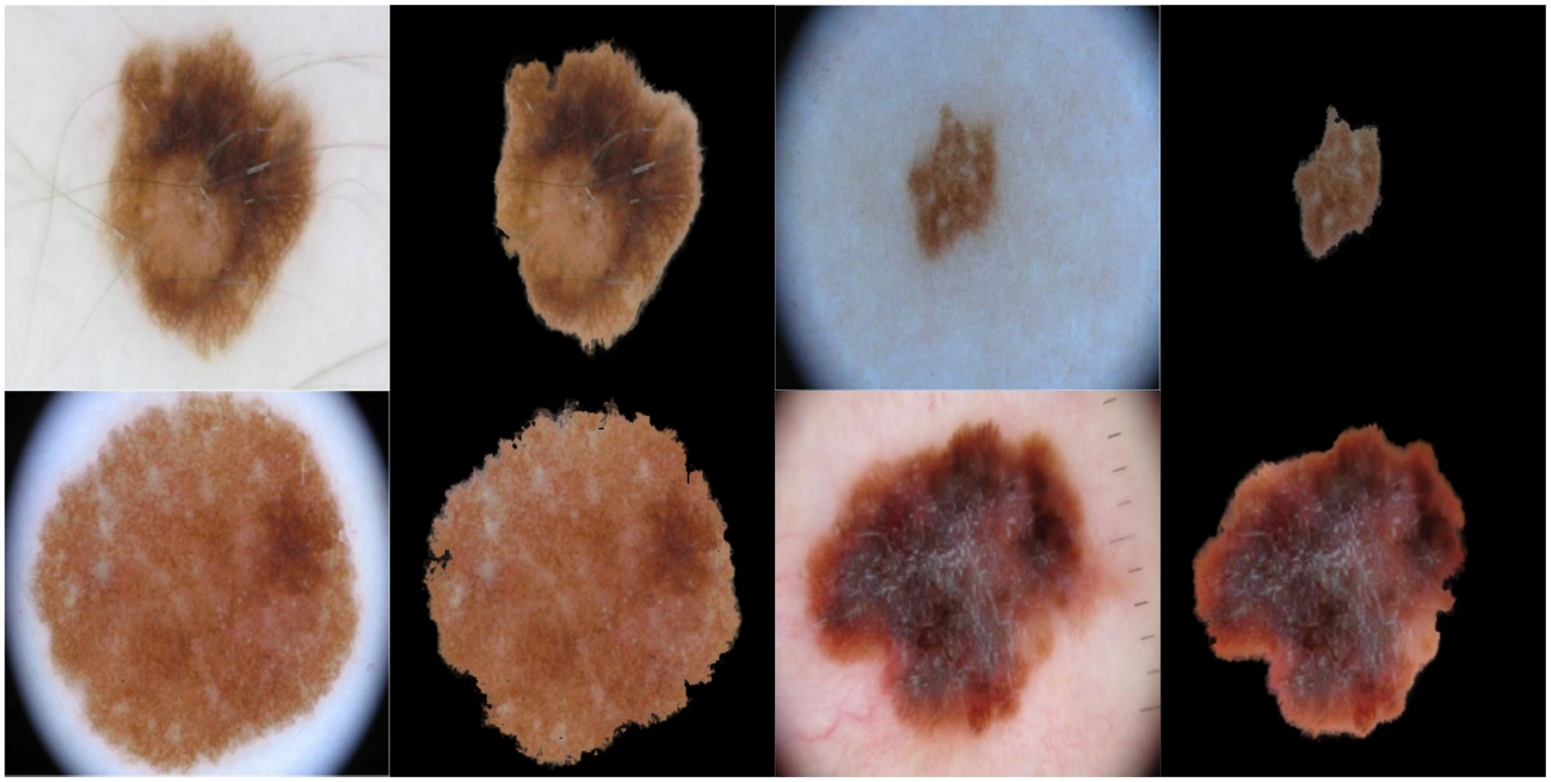
The original color image and its extracted lesion.

As we are going to measure asymmetry (especially when using Scale-invariant feature transform (SIFT)) and color variegation using the original color image, we would like to focus only on the extracted skin lesion rather than the background and any accompanying artefacts. This can be achieved by merging the original image (i.e. color image) and its corresponding alpha matte described above. Fig 2 shows some examples on original images and their extracted lesions using this process.

The proposed segmentation approach was applied on 204 randomly chosen skin lesion images (nevus: 175, melanoma: 29) extracted from the “ISIC 2018: Skin Lesion Analysis Towards Melanoma Detection grand challenge datasets” [33, 34]. The Dice coefficient [28] is used to measure the similarity between two images by finding the spatial overlap between two binary images, resulting in a value that lies between 0 (no overlap) and 1 (agree perfectly). The Dice coefficient can be defined as follows:

$$\begin{array}{c} D=\frac{2(A\cap G)}{A+G}\times 100\%\; \end{array}$$ (21)

where *A* is the algorithm output and *G* is the ground truth.

### Asymmetry

For measuring asymmetry we build a vector of three measurements that will be subsequently used to train and test a classifier (i.e. decision tree) as will be explained in more detail in the coming sections. Decision trees offer a structured way of decision making in pattern recognition and are characterized by an order of set nodes, such that each of the internal nodes is associated with a decision variable of one or more features [35]. The decision tree algorithm used in this paper is CART (Classification and Regression Trees) [36], which is represented as a binary tree (i.e. two branches). Tree nodes are expanded (i.e. depth) until all leaves are considered pure, that is when no further splits can be made.

#### SIFT based similarity

SIFT [37] is used as a sparse local descriptor where interest keypoints are detected in an image to describe invariant features (invariant to image scaling, rotation, and translation) in a local patch, such that an image will be represented as a collection of local feature vectors (shape descriptors) provided that a feature is a 128-dimensional vector representing a local region in the image. The goal of shape descriptors is to uniquely characterize the shape of the object to enable comparison of 2D object silhouettes. The group of feature vectors created by SIFT would thus represent the shape of the image. Using SIFT, we are detecting stable feature points in an image and then for each point a set of features that describe a small region around the point are provided, meaning that we are eventually extracting local information from digital images. Those extracted features are then used to match objects between different scenes.

We split the extracted lesions (Fig 2) vertically and horizontally across the centre into four equal halves, and use SIFT to measure the image similarity (showing correspondences) between each opposite half (top vs. bottom and right vs. left) using the 128-dimensional local feature vectors. The total similarity score is measured as *v*_*s*_ + *h*_*s*_, where *v*_*s*_ and *h*_*s*_ are the vertical and horizontal similarity, respectively. The greater the value the more similar the two halves of the skin lesion, and vice versa. Asymmetry means that the two halves are not similar. In measuring *v*_*s*_ and *h*_*s*_, we identify the best two matches for each keypoint using OpenCV’s brute-force matcher, where a feature descriptor on one image half will be matched with *all* feature descriptors on the other half and the best (closest) two matches will be returned based on the Euclidean distance. This is followed by the *ratio test* technique to remove any outliers (false positives) resulting from the brute-force matching step. Since we select the best two matches for each keypoint descriptor, ratio test considers a match to be good if the distance ratio between the first and second match is smaller than a specific empirical value (we chose it to be 0.7 which is a typical value for Lowe’s ratio). It should be emphasized that we only investigate the asymmetry of shape in this work, whilst dermatologists in general consider asymmetry in shape, color and structural (border) distribution.

**Fig 3:**
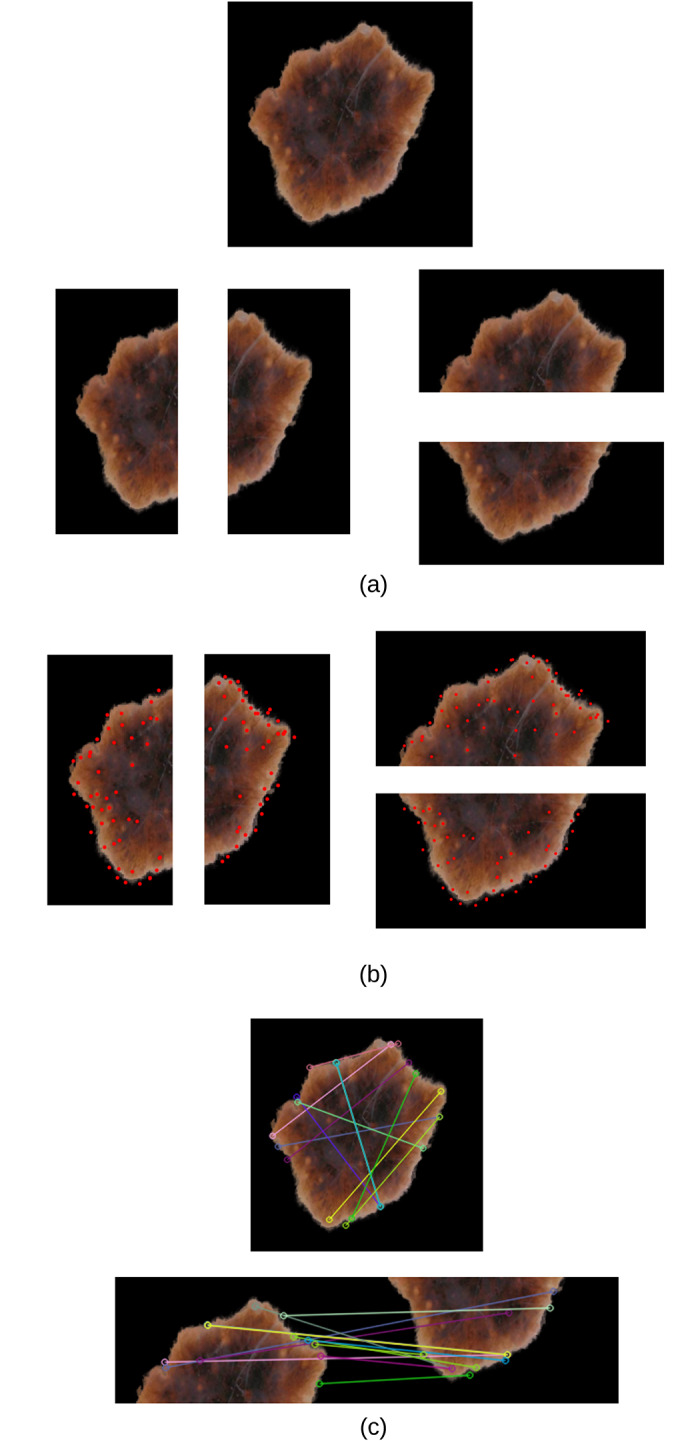
(a) Extracted skin lesion and its four halves along the vertical and horizontal axes (b) Keypoints (in red) of each half (c) Matching keypoints between each half (top figure: Left and right halves, bottom figure: Top and bottom halves).

Fig 3(a) shows an example of an extracted skin lesion and its four halves (vertical and horizontal halves), Fig 3(b) shows the keypoints of each half (in red), and Fig 3(c) shows the lines connecting the matching keypoints between each half. The similarity evaluates to 23 between the left and right halves and evaluates to 22 between the top and bottom halves. The total similarity in this skin lesion is the sum of the two similarity values (i.e. 23 + 22 = 45), making it tend to be more asymmetric and the likelihood to be a melanoma lesion.

#### Projection profiles

Projection profiles [38] are data structures that are used to store the number of foreground pixels when the image is projected over the X-Y axes. They are one-dimensional representations of a two-dimensional image content, and are considered as compact representations of images since many useful information is retained in projections. In this approach, symmetry is measured by projecting the segmented skin lesion in the *x* and *y* directions and then comparing their histograms. Assume we have a binary image of size *M* × *N* (M: height, N: width), the projection of the image onto a line can be obtained by partitioning the line into bins and finding the number of 1 valued pixels that are perpendicular to the bin. Horizontal and vertical projections can then be obtained by counting the number of 1 pixels from each bin in the horizontal and vertical directions, respectively.

The *horizontal projection* is the number of foreground (skin lesion) pixels in each row, and is defined as:

$$\begin{array}{c} H[i]=\sum_{j=0}^{m-1}B[i,j];\;0<i<N \end{array}$$ (22)

where *B*[*i*, *j*] is the pixel value at (*i*, *j*), and *H*[*i*] is the number of foreground pixels in the *i*^*th*^ horizontal row. Thus, for each horizontal line of pixels the number of foreground pixels are computed.

On the other hand, the *vertical projection* represents the number of foreground pixels in each column, and is defined as:

$$\begin{array}{c} V[j]=\sum_{i=0}^{n-1}B[i,j];\;0<j<M \end{array}$$ (23)

where *V*[*j*] is the number of foreground pixels in the *j*^*th*^ vertical column. The vertical projection of each column is thus computed.

**Fig 4:**
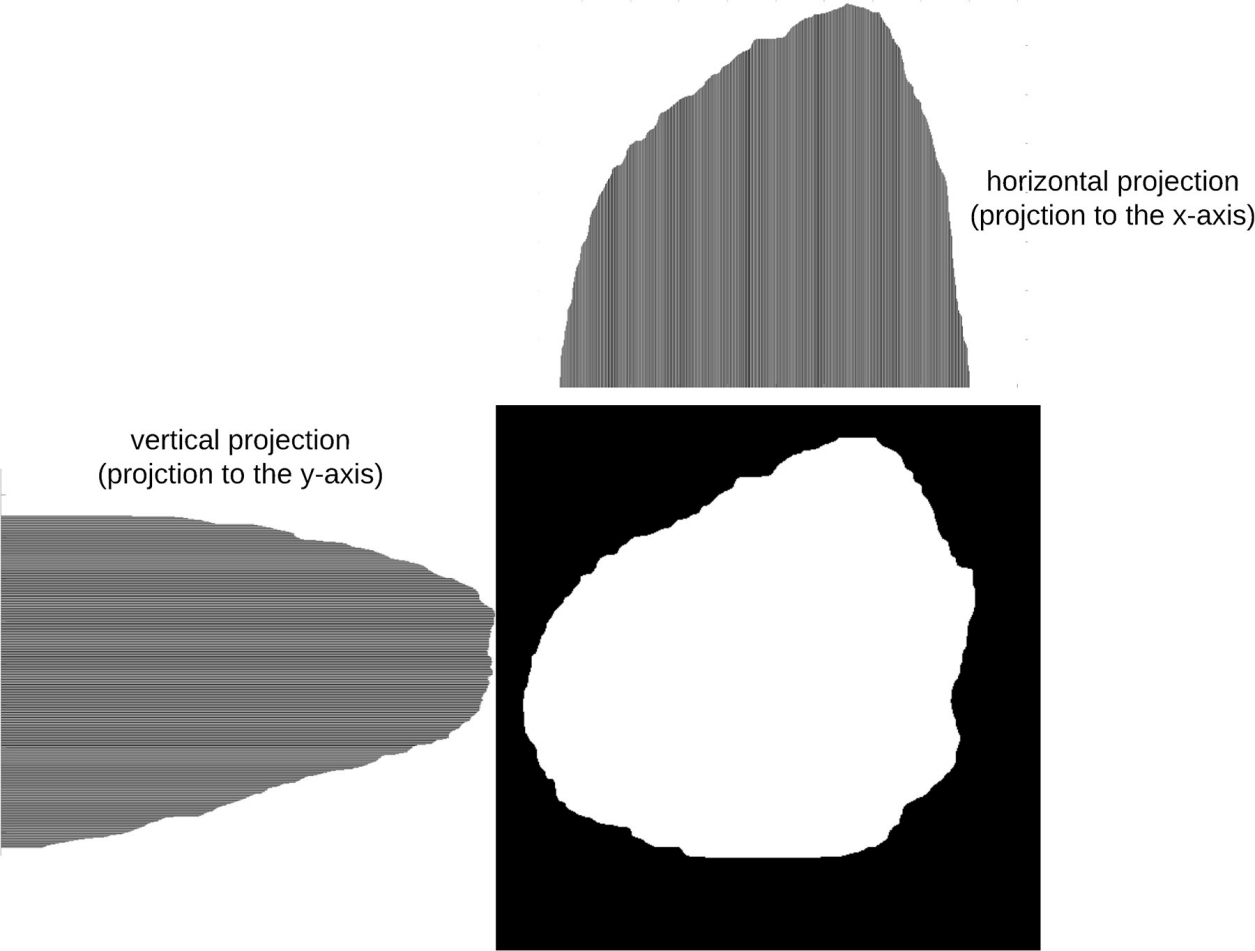
Histograms of horizontal and vertical projections.

The horizontal and vertical projection profiles can be represented as a histogram (Fig 4). The values of each histogram represent the density distribution of the skin lesion.

After finding the two histograms, they are compared using a *correlation* method. Let *I* denote the pixel intensity, *H*_1_(*I*) is the histogram which represents the number of pixels in the first image having pixel intensity *I*, and *H*_2_(*I*) is the histogram which represents the number of pixels in the second image having pixel intensity *I*. Using the correlation method, the two histograms are compared based on the following equations:

$$\begin{array}{c} d(H_{1},H_{2})=\frac{\sum_{I}^{}(H_{1}(I)-{H^{{}^{\prime }}}_{1})(H_{2}(I)-{H^{{}^{\prime }}}_{2})}{\sqrt{\sum_{I}^{}(H_{1}(I)-{H^{{}^{\prime }}}_{1})(H_{2}(I)-{H^{{}^{\prime }}}_{2})}} \end{array}$$ (24)

where,

$$\begin{array}{c} {H^{{}^{\prime }}}_{k}=\frac{1}{N}\sum_{J}^{}H_{k}(J) \end{array}$$ (25)

is the mean value of each pixel in image *k*, and *N* is the total number of histogram bins.

Symmetrical shapes (i.e. circle) evaluate to a correlation value of 1. The more asymmetrical the shape the less the correlation value.

#### Skewness

Image moments are useful in describing objects after the segmentation is carried out. They are scalar quantities that are used to capture the image’s signficant features. A measure of asymmetry in an image can be given by its skewness, which is a statistical measure of a distribution’s degree of deviation of the respective projection from symmetry. If the projection is symmetric with respect to the mean (origin), the corresponding skewness evaluates to *zero*. The *degree* of skewness can be determined using two third order moments: *M*_30_ and *M*_03_.

To map from the image domain to the momenta domain, the uniqueness theorem of the momenta [39] can be used which states that the momenta sequence (general moment) *M*_*pq*_ is determined by the density distribution function (i.e. skin lesion image). A *general/standard moment* *M*_*pq*_ of an image *f*(*x*, *y*) is defined as:

$$\begin{array}{c} M_{pq}=\sum_{x}^{}\sum_{y}^{}x^{p}y^{q}f(x,y) \end{array}$$ (26)

where *f*(*x*, *y*) are the graylevels of individual pixels, *p* and *q* are positive integers, and *r* = *p* + *q* is the *order* of the moment.

The third order moment is the skewness of distances between the pixels in the image and its geometrical centre, measuring the bias of the distribution of pixels. The direction of the skewness can be obtained from the sign of the result of the moment, such that when the moment is negative the distribution will bias towards the left of the centre, and when positive will bias towards the right of the centre [40].

The skewness of the horizontal and vertical projections can be defined as shown in Eqs 27 and 28, respectively.

$$\begin{array}{c} {skewness}_{H}=\frac{M_{30}}{\sqrt{{M_{20}}^{3}}} \end{array}$$ (27)

$$\begin{array}{c} {skewness}_{V}=\frac{M_{03}}{\sqrt{{M_{02}}^{3}}} \end{array}$$ (28)

### Color variegation

The identification of colors in skin lesions is considered a subjective task even for experienced dermatologists, which deems it necessary to develop an automatic objective approach to identifying colors in skin lesions. In this paper, we use the same rationale as in [19, 20] to determine the suspicious colors present in each skin lesion. However, we use the CIELab color space which is more representable of the human perception than the RGB color space. Moreover, we derive the suspicious colors CIELab values based on the color distribution of our dataset, making it more accurate in determining the suspicious colors. In addition, it is not clear how the colors in [19, 20] are derived (apart from the white, black and red colors in [20] where the standard RGB values are used), as the RGB value for the white color in [19] does not represent the actual color (another color is produced rather than the color of correspondence). Authors of those two studies used only one representative value for each suspicious color which might not be absolute (not representative enough) especially that we can have different levels (shades) of the same color (i.e. light brown). As opposed to those two studies, we use Minkowski distance instead of Euclidean distance.

Before attempting to find color variegation (The *C* feature in the ABCD rule) in the skin lesion, we convert the RGB image to the CIELab (or CIEL*a*b*) color space [41] since the RGB color space does not closely match the human visual perception, whereas the CIELab color space is designed to approximate/model the human vision (i.e. the *L* component closely matches the human perception of lightness) and contains in theory every single color the human eye can perceive, allowing it to exploit the characteristics of the human visual pereception better. Moreover, CIELab is considered more precise since the distance between colors using this color space corresponds to the perceived color distance. CIELab is device independent, meaning that the color model is based on the perception of the human eye and is designed to describe what colors look like regardless of what device they are displayed on.

To determine the range of color shades that represent the six suspicious colors of melanoma based on our image dataset, we extract and analyze the color palettes of *all* our images based on which we are able to determine the range of shades that would determine dark brown, light brown, … etc. In particular, each image in the dataset is represented in terms of its *dominant* colors which constitute the image’s palette, such that those dominant colors would be the best possible colors by which we can display the image with the least amount of error. Clusters of dominant colors (group of pixels) are formed using k-means clustering [42, 43], where each pixel in the image has a CIELab value associated with it. In this paper we set the number of clusters to 7 (6 suspicious colors + pure black) since we omit the pure black color (i.e. CIELab = [0, 0, 0]) in our measurement of the number of suspicious colors as it most likely belongs to the background (skin).

The process consists of applying k-means with a specific number of clusters (i.e. dominant colors) which is equal to the number of colors the color palette will be composed of. Each pixel color is then affected to the nearest cluster centroid according to the Euclidean distance. K-means minimizes the within-cluster sum of squared distances (i.e. Euclidean distance) between the centroid and the other pixels in the cluster. Using Euclidean distance in the CIELab space is uniform with difference perceived by the eye.

**Fig 5:**
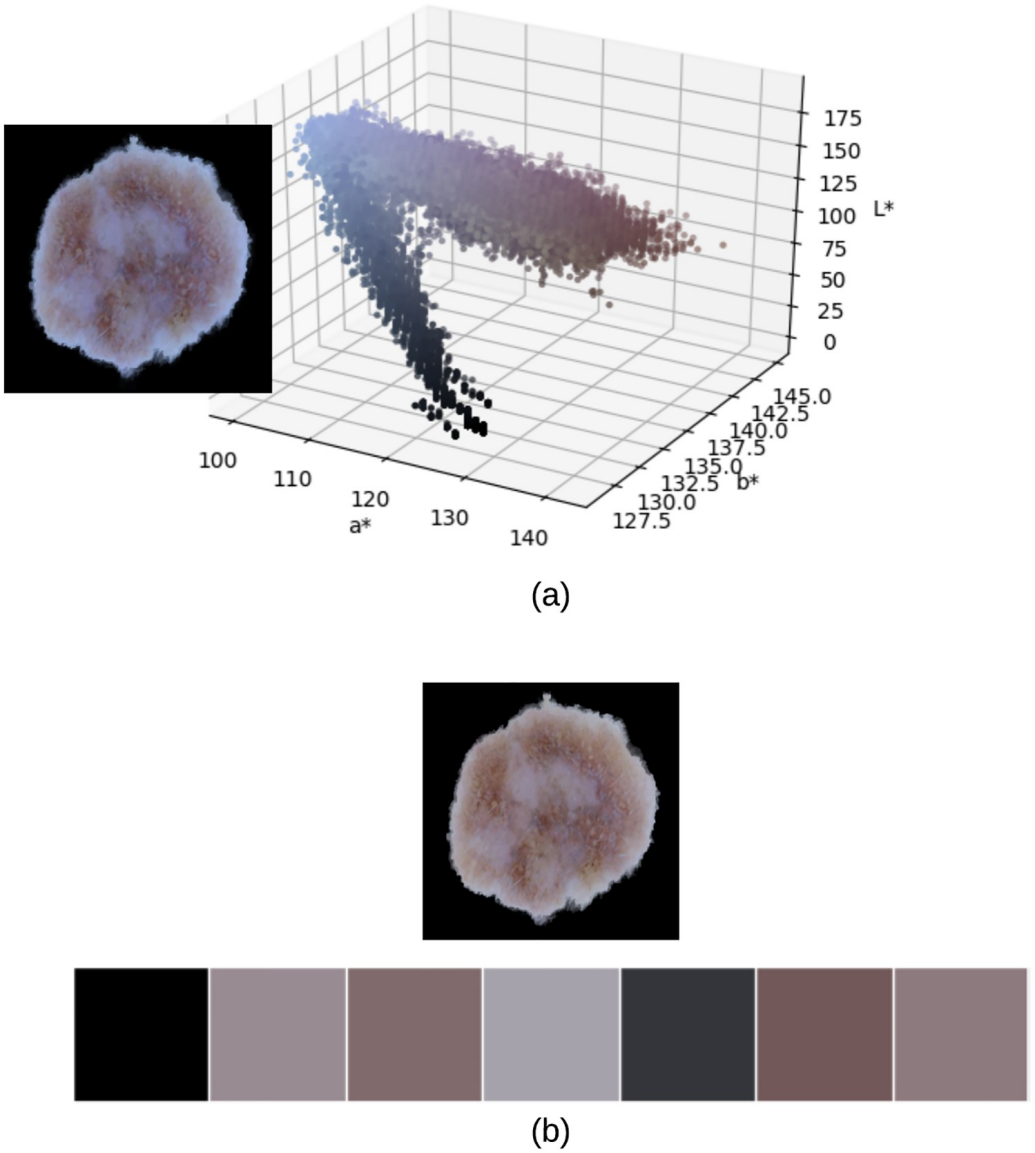
(a) 3D scatter plot of skin lesion image pixels in CIELab color space (b) Skin lesion color palette showing the 7 dominant colors in the image (the first color which represents the pure black color will be omitted from our color variegation measurement).

K-means finds the dominant colors in an image through an iterative corrective process, where colors can be thought of as points in the color space cloud that we aim to cluster around some mean (dominant color). K-means starts with a random palette of seven dominant colors {1, 2, …, *k*} as the starting point, where *k* is the number of clusters representing the dominant colors. Each pixel is then assigned a color label of the nearest dominant color. Image pixels in this case are thus grouped by their dominant color value. New averages are then computed to update the cluster centers. If image pixels belong to the same clusters for two successive iterations, the process is considered finished and the final color palette is formed. Fig 5 shows a skin lesion image and its 3D scatter plot representing the different pixel colors in CIELab color space, in addition to the lesion’s color palette resulting from k-means clustering with *k* = 7. The CIELab values of the color palette from left to right are: [0, 0, 0], [59.263, 6.519, -1.826], [47.051, 9.465, 2.307], [67.222, 3.355, -4.661], [22.237, 0.779, -3.284], [40.018, 10.696, 4.107], [53.075, 8.206, -0.027].

Representing the image in terms of its dominant colors is a more realistic approach when working on color variegation; for instance the image shown in Fig 5 is composed of 1480 unique colors, which makes it very difficult in determining the range of color shades that would represent the 6 suspicious colors of melanoma.

Analyzing the images used in our experiments, the suspicious CIELab color ranges (i.e. [*min*] − [*max*]) are determined as shown in Table 4. For the suspicious colors white, red and blue-gray, since they are not expressed in our dataset we use their standard CIELab color values. Skin lesions which have any of their pixel CIELab color values belonging to the range of black, dark brown, or light brown color values are considered to possess those colors. For the colors of white, red and blue-gray, a skin lesion is considered to possess one of those colors if the (i) Minkowski distance between the pixel color and any of the aforementioned colors is less than a threshold *T*, measured as being the half of the Minkowski distance between the two extremes of colors (white and black), which evaluates to 50 (ii) pixels that belong to the suspicious color represent more the 5% of the skin lesion pixels.

**Table 4:** CIELab melanoma suspicious color values.

Color	CIELab
Black	[0.06, 0.27, 0.10] − [39.91, 30.23, 22.10]
Dark brown	[14.32, 6.85, 6.96] − [47.57, 27.14, 46.81]
Light brown	[47.94, 11.89, 19.86] − [71.65, 44.81, 64.78]
White	[100, 0, 0]
Red	[54.29, 80.81, 69.89]
Blue-gray	[50.28, −30.14, −11.96]

Minkowski distance is considered a generalization of the Euclidean and Manhattan distances [44] and is defined as [45]:

$$\begin{array}{c} d_{m}(x_{i},x_{j})=\sqrt[{p}]{\sum_{k=1}^{n}{\left|x_{ik}-x_{jk}\right|}^{p}} \end{array}$$ (29)

where *p* ≥ 1 is a real number. The distance represents the Manhattan distance and the Euclidean distance when *p* = 1 and *p* = 2, respectively.

The advantage of using Minkowski distance is that mathematical results can be shown for the whole class of distance functions, and users can adapt the distance function to suit the needs of the application by modifying the Minkowski parameter *p*, which is set to *p* = 3 in this paper. Based on the above, the image in Fig 5 is composed of 3 suspicious colors.

### Diameter

To measure the diameter of the skin lesion we utilize Feret’s diameter [46, 47], which is the distance between two parallel tangents at the contour of the object (i.e. skin lesion) that are located on opposite sides of the object at an arbitrary selected angle. The maximum Feret diameter of an object is the distance between its two furthest points measured in a given direction. The average value over many orientations can also be used, meaning that Feret’s diameter can be referred to as the average distance between two tangents in the opposite sides of the object parallel to some fixed direction. In this paper we use the maximum Feret diameter as our diameter measure. Finding the Feret’s diameter of some object shape is a commonly used measure in shape analysis.

Feret’s diameter is also called caliper diameter since the measurement involves placing the object for which we want to find the diameter inside the jaws of a caliper, with the caliper oriented at some specified angle (i.e. 0°, 45°, 90°, 135°). The jaws are then closed on the object tightly while maintaining the angle. The distance between the jaws is the Feret diameter at angle (direction) *θ*. In terms of digital images, this is made by isolating the corner pixels of the object’s perimeter and taking the maximum distance between each corner pixel to all other corner pixels, meaning that Feret’s diameter of an object in direction *θ* is the projection of the object on the axis oriented in direction *θ*. It should be emphasized that Feret’s diameter is based on the binary 2D image of the object. The main advantage of using Feret’s diameter over other measures is due to its correspondence with the real physical diameter of the object. In other words, it corresponds to the length that would be measured if we handle the object between the teeth of a caliper.

**Fig 6:**
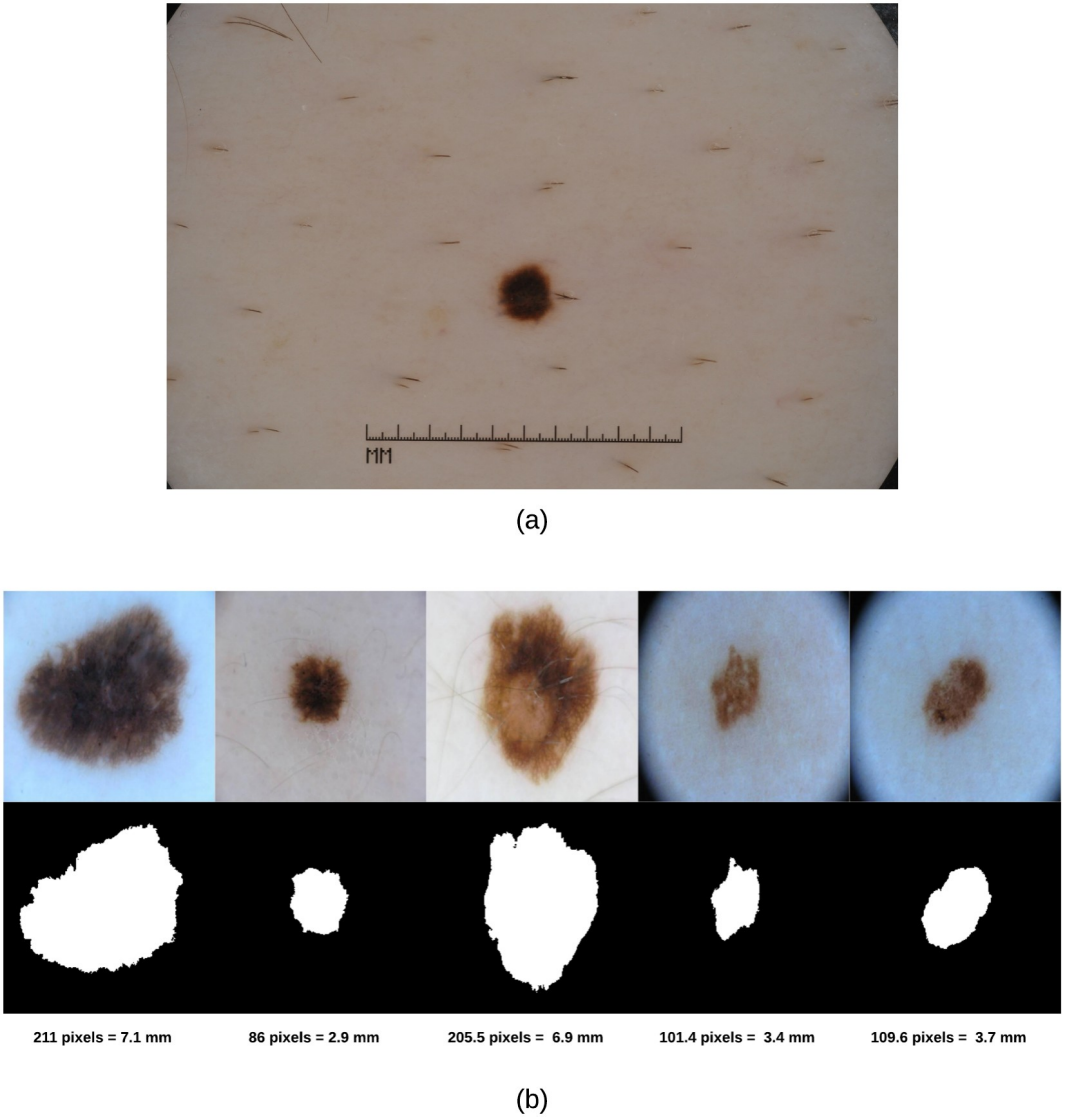
(a) Skin lesion image with a ruler used to spatially calibrate our test images and deduce the pixels/mm value (b) Skin lesions’ Feret’s diameter (in pixels and millimeters).

As the skin lesion diameter in the real world is measured in millimeters (mm) and our diameter results are returned in pixels, we need to represent our diameter results in terms of the standard unit (mm); this can be made using spatial calibration (geometric correction) which involves calibrating the image against a known value (i.e. mm) and then applying such calibration to the uncalibrated image (i.e. in pixels). The idea is thus to represent the diameter in units rather than pixels. An image produced in units is called a spatially calibrated image. However, to conduct such calibration one needs to know the original measure in real world and then map that to pixels. Since we do not have the original real world measures available, we used an image in our dataset (i.e. ISIC dataset) that has a ruler displayed (Fig 6(a)) to get an estimate on the skin lesion measure in millimeters and deduce from that how many pixels would be in 1 mm. The image was also zoomed in to better reflect the sizes of the skin lesions in our test images. Doing such calibration, we found that for our 256 × 256 images we had 29.7 pixels/mm. Fig 6(b) shows samples of skin lesion images, their segmentations and corresponding Feret’s diameter values.

After applying the methods explained above, we use the extracted features to derive a decision on the asymmetry, color variegation (i.e. number of suspicious colors), and diameter on the skin lesion images fed to our system.

## Results and discussion

**Fig 7:**
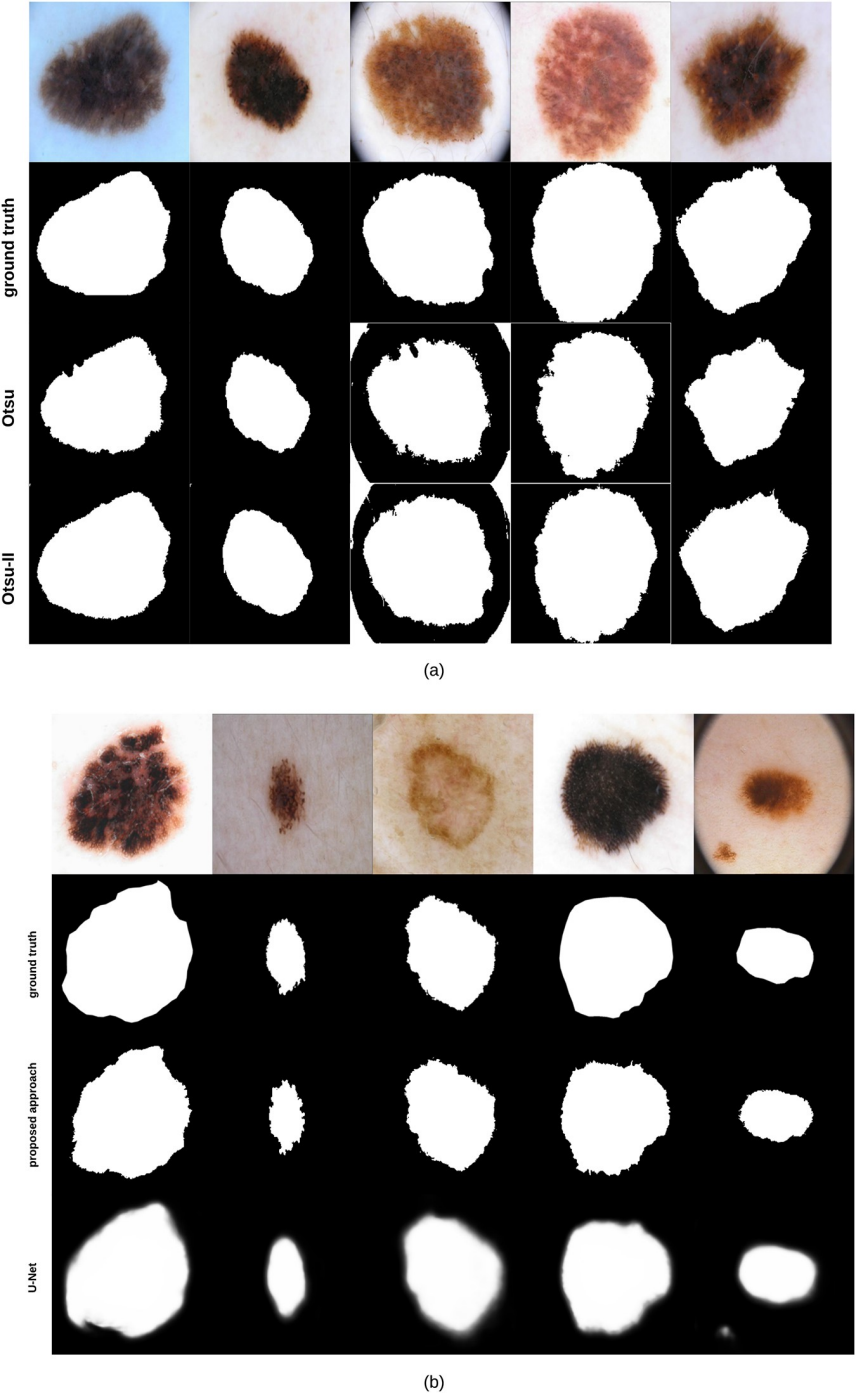
(a) Skin lesion images segmented using Otsu and Otsu-II methods, along with their corresponding groundtruth (b) Skin lesion images segmented using our proposed segmentation approach and U-Net, along with their corresponding groundtruth.

Applying *Otsu-II* (the improved Otsu thresholding version we propose in this paper) and the original Otsu approach on the 204 images lead to a Dice similarity coefficient value of 87.7% and 82.5%, respectively. Fig 7(a) shows some examples on segmentation results obtained by Otsu and Otsu-II methods, along with the groundtruth of each corresponding image. As can be noticed, Otsu-II is able to improve the area coverage of the skin lesion.

It should be emphasized that we tested another adaptation of Otsu’s method where we kept *P*_*L*_(*k*) and *P*_*S*_(*k*) from Eqs 6 and 7 in Eqs 17 and 18 (i.e. Otsu-II), resulting in the new within and between class variances shown in Eqs 30 and 31, respectively, which we refer to here as Otsu-II′:

$$\begin{array}{c} \sigma_{W}^{2}(k)=D_{L}(k)\times P_{L}(k)\times \sigma_{L}^{2}(k)+D_{S}(k)\times P_{S}(k)\times \sigma_{S}^{2}(k) \end{array}$$ (30)

$$\begin{array}{c} \sigma_{B}^{2}(k)=D_{L}(k)\times P_{L}(k)\times {\left(\mu_{L}(k)-\mu_{T}(k)\right)}^{2}+D_{S}(k)\times P_{S}(k)\times {\left(\mu_{S}(k)-\mu_{T}(k)\right)}^{2} \end{array}$$ (31)

Having three Otsu related methods, we expanded our evaluation on two more types of datasets. One dataset contained skin lesions that cover most of the image (i.e. large lesions) and another set where the skin lesion comprises a small region of the image. The dataset used with Otsu and Otsu-II methods above contained a mix of such lesions (i.e. mixed). The datasets that contained images with large and small skin lesions were composed of 129 and 162 images, respectively.

The Dice similarity of the three Otsu related methods on the different datasets is summarized in Table 5. The poor performance of Otsu-II′ on small images was due to the fact that it was not robust to artifacts (i.e. hair, ruler) that were more apparent in images with small skin lesions.

**Table 5:** Dice similarity of Otsu related methods on three datasets.

Dataset	Otsu	Otsu-II	Otsu-II′
Small	71.2%	74%	49.6%
Large	84.6%	88.7%	88.5%
Mixed	82.5%	87.7%	85.6%

To evaluate the performance of our segmentation approach (Fig 1) against state-of-the-art methods, we compare our approach with U-Net [48], an end-to-end encoder-decoder network firstly used in medical image segmentation and has also been utilized in skin lesion segmentation in dermoscopic images [49, 50]. U-Net is trained on 1935 dermoscopy images along with their corresponding groundtruth response masks. Images used to train U-Net were resized to 256 × 256 pixels, and the model was trained for 20 epochs on a Tesla P100 GPU. Training the model took 27.1 minutes and testing it on the 204 images took 18.1 seconds. Fig 7(b) shows some samples of test images, their corresponding groundtruth, and the results using our proposed segmentation approach and U-Net. The average Dice similarity of the 204 test images evaluates to 88% and 76.2% for our proposed segmentation approach and U-Net, respectively. Visual results (Fig 7(b)) show that our approach is able to detect the fine structures of skin lesion borders—a crucial factor when detecting skin lesion border irregularity (i.e. B feature in the ABCD rule)—better than U-Net, a feature that U-Net seems to lack. The results of U-Net as can be noticed are a bit blurry. Such blurriness can be removed by applying a thresholding technique, but we kept the original results of U-Net as-is for comparison purposes.

**Fig 8:**
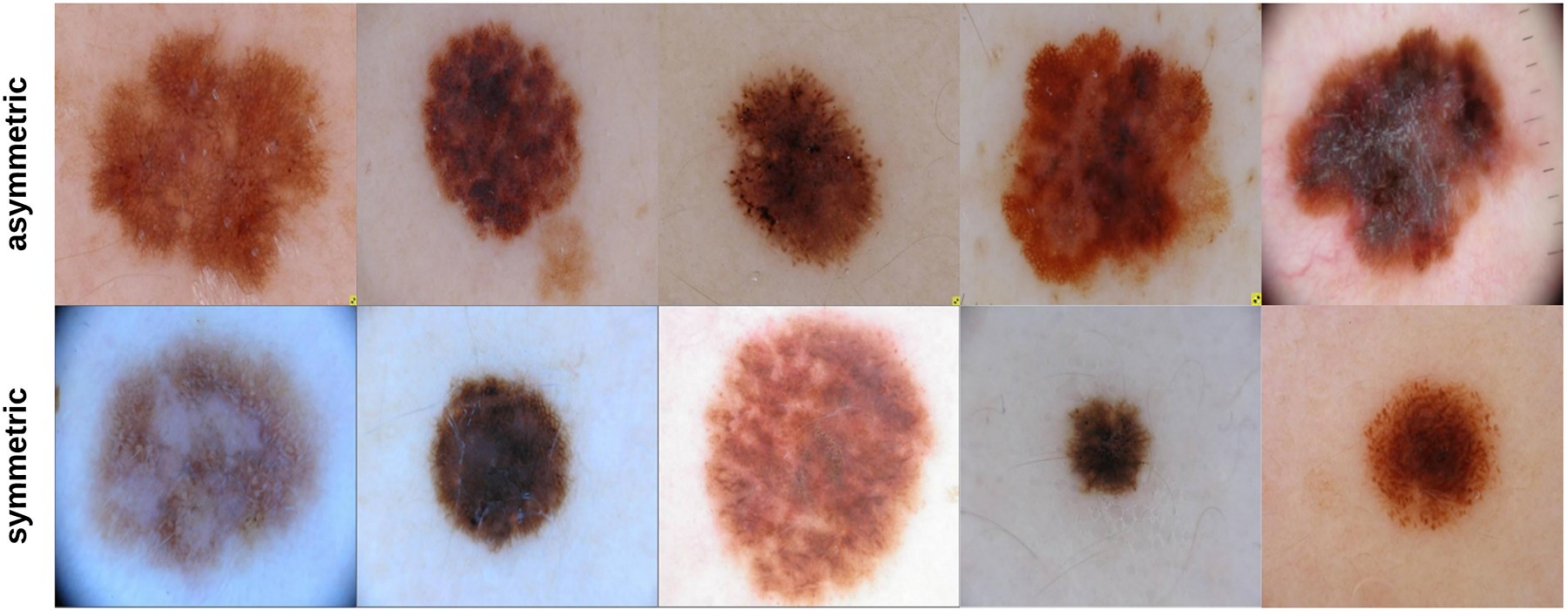
Samples of symmetric and asymmetric images labeled by the dermatologist.

204 skin lesion images were sent to a dermatologist (Dr.Sally O’Shea) to label as *symmetric* or *asymmetric* based on shape (most of the skin lesions contained 1 suspicious color and a diameter larger than 150 pixels—5.1 mm). Of the 204 images labeled by the dermatologist, 35 images were classified as being symmetric and 169 images as asymmetric. Fig 8 shows some samples of symmetric and asymmetric images. The extracted asymmetry features/measurements (SIFT based similarity, projection profiles, and skewness) have been used to train and test a *decision tree* on an 80: 20 ratio. That is, 80% of the data was used for training the decision tree and 20% of the data was used for testing the decision tree. 136 asymmetric and 28 symmetric images were used for training the decision tree. 33 asymmetric and 7 symmetric images were used for testing the decision tree. After training the decision tree, 30 and 2 asymmetric and symmetric images were predicted correctly, respectively, meaning that an 80% accuracy has been obtained. The decision tree as can be noticed performed better on asymmetric images; this can be due to having more asymmetric images in the training data. But, in general, we believe that more data samples which are also balanced (number of asymmetric and symmetric samples are the same) could improve the results significantly. This requires much labor work (i.e. labeling by dermatologists) and is a topic of interest we would be willing to explore in future studies.

**Fig 9:**
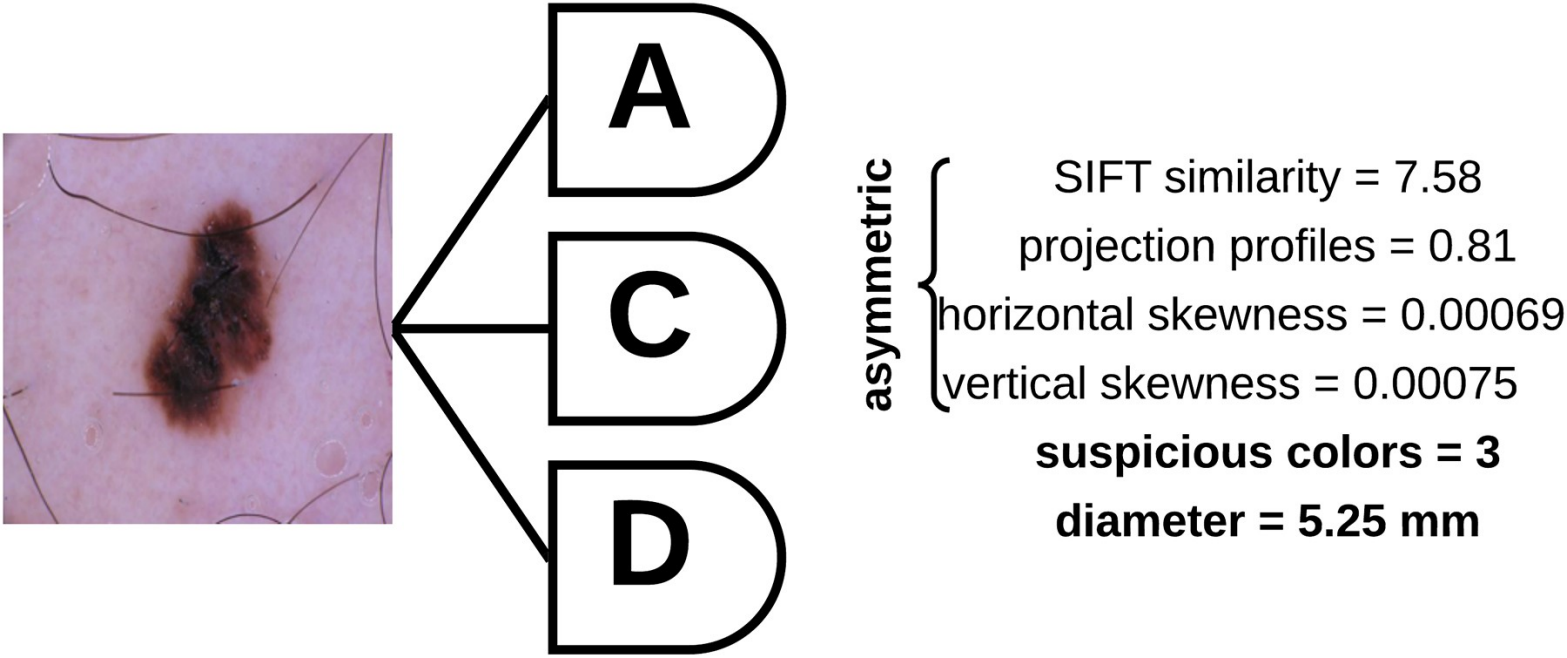
Skin lesion asymmetry, color variegation, and diameter features extracted using the proposed approach.

To determine the asymmetry, color variegation, and diameter of an unknown (test) sample, the asymmetry features (SIFT based similarity, projection profiles, and skewness) are extracted and fed to a decision tree which is used to predict whether the skin lesion is symmetric or asymmetric based on the extracted measures. The number of suspicious colors in the skin lesion are then determined based on the values shown in Table 4, and the diameter is then measured based on Feret’s diameter explained above. Fig 9 shows a skin lesion and its extracted features using the proposed approach.

## Conclusion

To overcome the subjectivity in measuring asymmetry and color variegation, and to avoid missing melanoma in the early diagnosis due to possessing a diameter less than 6mm, an objective approach has been proposed where the extracted asymmetry measurements were used to train a decision tree which was then utilized for predicting the asymmetry of new skin lesion images. The range of the CIELab color space colors that demonstrate the suspicious colors of melanoma have been derived, and the diameter has been found using the Feret’s diameter method. The approach is able to predict asymmetry with 80% accuracy and measure color vriegation and diameter objectively. The proposed Otsu improvement (Otsu-II) outperforms the original both Otsu and Otsu-II′ in skin lesion segmentation, and when combined with the pre/post processing steps to represent our segmentation approach, this provides better segmentation results than U-Net. In summary, the contribution of our work involves proposing a segmentation approach that outperforms standard segmentation approaches, an asymmetry measure composed of a vector of SIFT based similarity, projection profiles, and skewness which can then be automatically determined using a trained decision tree, and objective approaches to determine the number of suspicious colors in a skin lesion along with its diameter. The measures can be incorporated in a desktop computer vision system where clinicians can feed the system with dermoscopic images and obtain an objective evaluation on those measures. In future work we aim at using a more balanced dataset with more symmetric and asymmetric samples (requires more laborious work), as this could improve the decision tree prediction accuracy. Enhancing Otsu-II and making it more robust to different artifacts will also be investigated. Moreover, different machine learning approaches will be analyzed.
